# Supplementary material for: Autonomous DNA nanostructures instructed by hierarchically concatenated chemical reaction networks
Source: Nat Commun. 2021 Aug 26;12:5132. doi: 10.1038/s41467-021-25450-5 (PMC8390752; doi:10.1038/s41467-021-25450-5)
Supplement: Supplementary file 1 — Supplementary Information [file 41467_2021_25450_MOESM1_ESM.pdf]

Supplementary Information for

Autonomous DNA nanostructures instructed by hierarchically concatenated chemical reaction networks

Jie Deng[1,3]* and Andreas Walther[1,2]*

[1] A^{3}BMS Lab, Department of Chemistry, University of Mainz, Duesbergweg 10-14, 55128 Mainz, Germany

[2] Cluster of Excellence livMatS @ FIT – Freiburg Center for Interactive Materials and Bioinspired Technologies, University of Freiburg, Georges-Köhler-Allee 105, D-79110 Freiburg, Germany

[3] Present address: Department of Cancer Biology, Dana-Farber Cancer Institute, Harvard Medical School, Boston, MA 02115, USA

E-mail: andreas.walther@uni-mainz.de; jie_deng@dfci.harvard.edu

Materials

Instrumentation: ThermoMixer (Eppendorf), thermocycler Tpersonal (Analytik Jena), INTAS *CHEMOSTAR touch* fluorescence imager (INTAS Science Imaging), gel electrophoresis chambers (biostep), Enduro power supply 300 V (Labnet International, Inc.), ScanDropR UV-VIS spectrometer (Analytik Jena), confocal laser scanning microscopy (Leica TCS SP8, Mannheim), and Spark® multimode microplate reader (Tecan).

Reagents: T4 DNA ligase (HC, 20 Weiss units (WU) µL^{-1}, recombinant *E. coli* strain) was supplied by Promega and BsaI-HF®v2 (20 units (U) µL^{-1}, *NEB #R3733*) and 10× CutSmart® Buffer were ordered from New England Biolabs (*NEB*). ATP solution (10 mM in 1 mM Tris-HCl pH 7.5) was purchased from Invitrogen. Agarose low EEO was supplied by PanReac AppliChem. Gene ruler 50 bp DNA ladder (ready to use), and DNA gel loading dye (6×) were supplied by ThermoFisher Scientific. Ethylenediaminetetraacetic acid disodium salt dihydrate (EDTA, biology grade) was supplied by CALBIOCHEM. Sodium chloride (NaCl, 99%), hexadecane (99%), tris (hydroxymethyl)aminomethane hydrochloride pH 8.0 (Tris-HCl), trizma base, magnesium chloride solution (2 M in H_2O, BioUltra), BRAND® cover glass (0.13-0.17 mm, No. 1, L × W 24 mm × 60 mm, rectangular), and 24-well silicone isolators (press-to-seal, 2 mm diameter, 0.6 mm depth) were ordered from Sigma-Aldrich. Acetic acid glacial (ACS, Reag. Ph. Eur. Analytical reagents) was supplied by VWR Chemicals. RotiR-GelStain (1,1'3,3',5,5'6,6'-Octamethyl-2,2'-spiro(2,3-dihydro-1H-Benzimidazol) was supplied by Carl Roth. All oligonucleotides purified by high-performance liquid chromatography were supplied by Integrated DNA Technologies Inc., and the sequences and modifications are shown below.

Buffer compositions

T4 DNA Ligase Storage Buffer (Promega): 10 mM Tris-HCl (pH 7.4 at 25 °C), 50 mM KCl, 1 mM dithiothreitol (DTT), 0.1 mM EDTA, 50% glycerol.

BsaI-HF®v2 storage buffer (NEB): 10 mM Tris-HCl, 200 mM NaCl, 1 mM DTT, 0.1 mM EDTA, 200 µg/mL BSA, 50% glycerol.

NEB CutSmart® Buffer: 50 mM potassium acetate, 20 mM Tris-acetate, 10 mM magnesium acetate, 100 µg mL^{-1} BSA.

Annealing Buffer: 10 mM Tris-HCl (pH 8.0), 50 mM NaCl.

TAE Buffer: 40 mM Tris, 20 mM acetic acid, 1 mM EDTA.

Quenching Buffer: 200 mM EDTA, 10 mM Tris-HCl (pH 8.0), 50 mM NaCl.

Tris Acetate-EDTA (TAE)/Mg^{2+}: 40 mM Tris, 20 mM acetic acid, 1 mM EDTA, 12.4 mM $MgCl_2$, pH 8.0.

Milli-Q water was used throughout of this study.

Supplementary Table 1: Oligonucleotide sequences used, with their name in individual Figure, the sequence codes used for ordering at IDT.

	**Name**	**Oligonucleotide sequence (5’-3’)**	**#nt**
**Fig. 2-4, 6, 7, S2**	Complex 1	CATGAGAATTCCATTCACGGTCTCT	25
		/5Phos/GATTAGAGACCGTGAATGGAATTCTCATG	29
**Fig. 2- 4**	Substrate 1	CGGATTGGTATTGTATTA	18
		/5Phos/AATCTTTAATACAATACCAATCCGATT	27
	Input 1	/5Phos/AATCAATCGGA	11
	Input 2	GTATTAAA	8
**Fig. 2**	Substrate 2	/5IAbRQ/CGGATTGGTATTGTATTA/3Cy5/	18
		/5Phos/AATCTTTAATACAATACCAATCCGATT	27
**Fig. 3**	Substrate 3	TTGGTATTGTC/3Cy5/	11
		/5IAbRQ/GACAATACCAATCCG	15
**Fig. S2**	Substrate 4	CGGATTGGATCTATTGTATTA	21
		/5Phos/AATCCTTTAATACAATAGATCCAATCCGATT	31
	Substrate 5	TTGGATCTATTGTC/3Cy5/	14
		/5IAbRQ/GACAATAGATCCAATCCG	18
	Input 3	/5Phos/AATCAATCGGAT	12
	Input 4	TGTATTAAAG	10
**Fig. 4, 6, 7**	S 1	CTCAGTGGACAGCCGTTCTGGAGCGTTGGACGAAACT	37
**Fig. 4**	S 2	TTGGTATTGTCTGGTAGAGCACCACTGAGAGGTA	34
**Fig. 4, 6, 7**	S 3	/5ATTO488NN/TCCAGAACGGCTGTGGCTAAACAGTAACCGAAGCACCAACGCT	43
	S 4	CAGACAGTTTCGTGGTCATCGTACCT	26
	S 5	CGATGACCTGCTTCGGTTACTGTTTAGCCTGCTCTAC	37
**Fig. 4**	Inhibitor 1	GACAATACCAATCCG	15
	Activator (=Output 1)	CGGATTGGTATTGTATTA	18
	Deactivator	/5Phos/AATCTTTAATACAATACCAATCCGATT	27
**Fig. 6, 7**	S 2*	/5Phos/AATCTTATGTTTGTATTGTCTGGTAGAGCACCACTGAGAGGTA	43
	Inhibitor 2	GACAATACAAACAT	14
	Input 5	AAACATAA	8
**Fig. 7**	S 1-#2	ACGGATCCTGATAGCGAGACCTAGCAACCTGAAACCA	37
	S 2-#2	/5Phos/AATCGTATGTTTGTATTCTGTCCTACTCGTGGATCCGTGGTAT	43
	S 3-#2	/5ATTO647NN/AGAATTGCGTCGTGGTTGCTAGGTCTCGCTATCACCGATGTG	42
	S 4-#2	GACAGTGGTTTCACCTGAACGATACC	26
	S 5-#2	CGTTCAGGACGACGCAATTCTCACATCGGACGAGTAG	37
	Inhibitor 3	CAGAATACAAACAT	14
	Input 6	AAACATAC	8

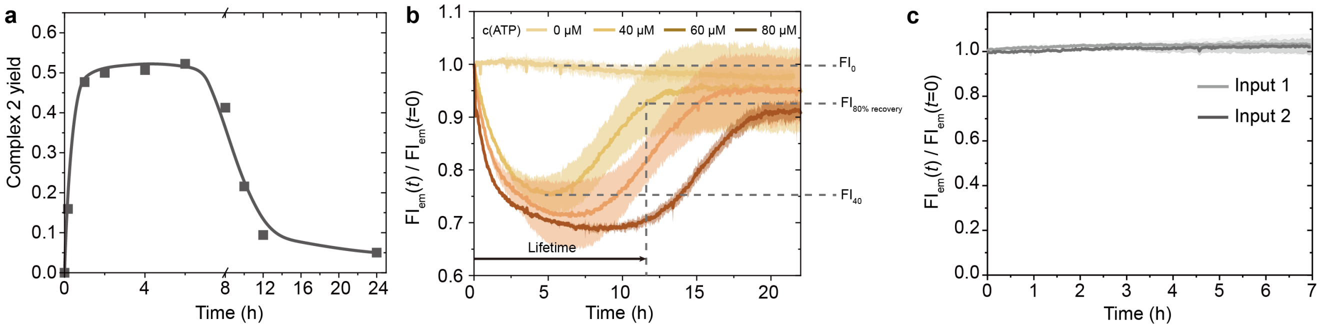

Supplementary Fig. 1. ATP-fueled transient DSD. (**a**) Time-dependent yield of Complex 2 in the ATP-fueled transient DSD quantified from AGE data using the equation above. Line is a guide to the eye. (**b**) Illustration of data treatment for determine the lifetime for ATP-fueled transient DSD. FI represents fluorescence intensity. (**c**) ATP-fueled DSD for transient allosteric switch triggered by Input 1 or Input 2. Conditions: (**a**) 25 °C, 20 μM Complex 1, 5 μM Substrate 1, 10 μM Input 1, 10 μM Input 2, 0.8 WU μL^{-1} T4 DNA ligase, 1.5 U μL^{-1} BsaI, and 40 μM ATP. (**c**) 25 °C, 20 μM Complex 1, 5 μM Substrate 2, 0.8 WU μL^{-1} T4 DNA ligase, 1.5 U μL^{-1} BsaI, 40 μM ATP, and 10 μM Input 1 or 10 μM Input 2.

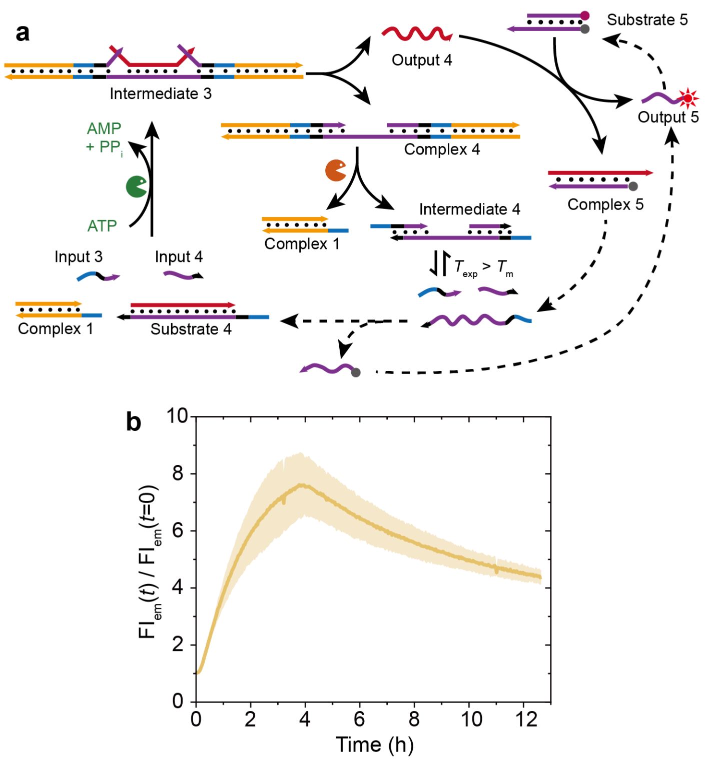

Supplementary Fig. 2. ATP-fueled transient DSD cascades at 37 °C. (**a**) Schematic representation of ATP-fueled transient DSD cascades. (**b**) Time-dependent FI for monitoring ATP-fueled transient DSD cascades by substrate 5 with one equivalent amount of quencher strand. Conditions: 37 °C, 20 µM Complex 1, 5 µM Substrate 4, 1 µM Substrate 5 (with one equivalent amount of quencher strand), 10 µM Input 3, 10 µM Input 4, 0.8 WU μL^{-1} T4 DNA ligase, 1.5 U μL^{-1} BsaI, and 40 µM ATP.

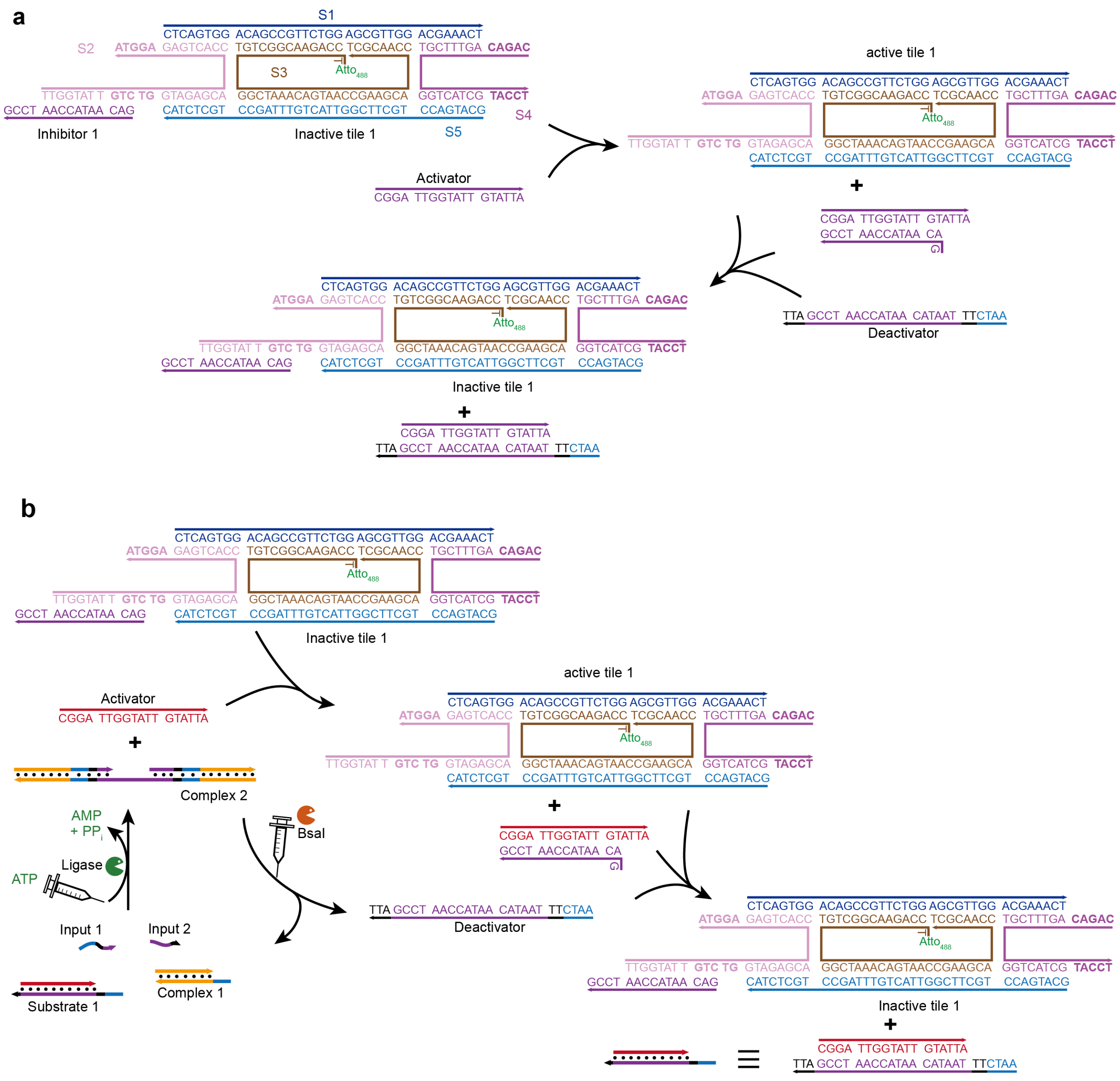

Supplementary Fig. 3. Illustration of reversible tile activation and deactivation reactions. (**a**) Schematic representation of Inactive tile 1 annealed from S 1, S 2, S 3, S 4, S 5, and Inhibitor 1, and its activation and deactivation by Activator and Deactivator, respectively. (**b**) Schematic representation of ATP-powered ligation induced tile activation and later on BsaI restriction induced tile deactivation.

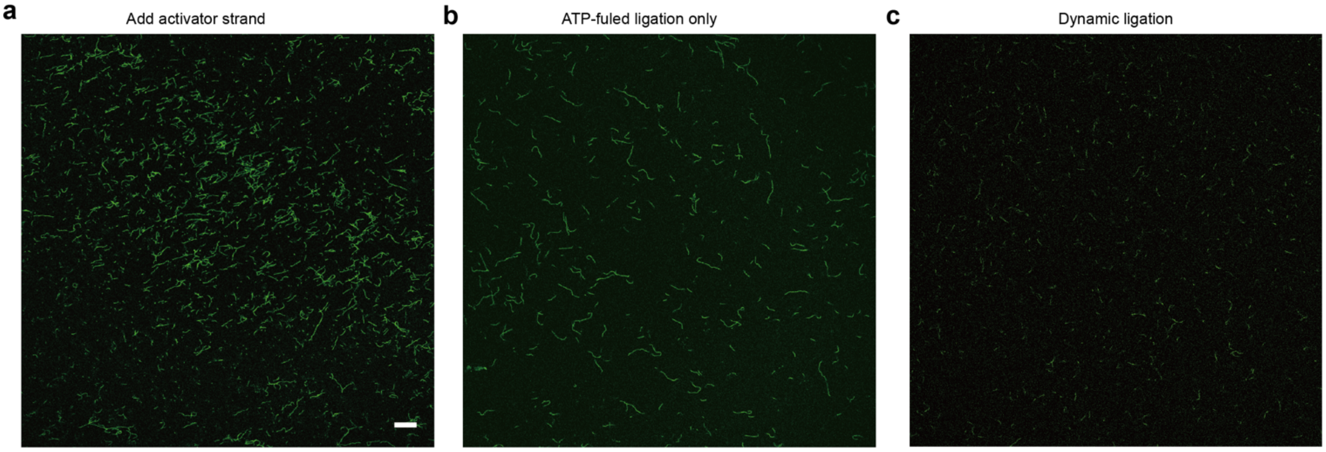

Supplementary Fig. 4. Zoomed-out confocal laser scanning microscopy (CLSM) images of assembled DNA nanotubes (DNTs) to shower larger field of view. (**a**) Representative CLSM image of DNT self-assembly activated by adding Activator strand (30 min reaction time at 25 °C). (**b**) Representative CLSM image of DNT self-assembly triggered by ATP-fueled ligation only (2 h reaction time at 25 °C). (**c**) Representative CLSM image of DNT self-assembly in an enzymatic reaction network of concurrent ATP-powered ligation and restriction (2 h reaction time at 25 °C). Scale bar = 10 µm.

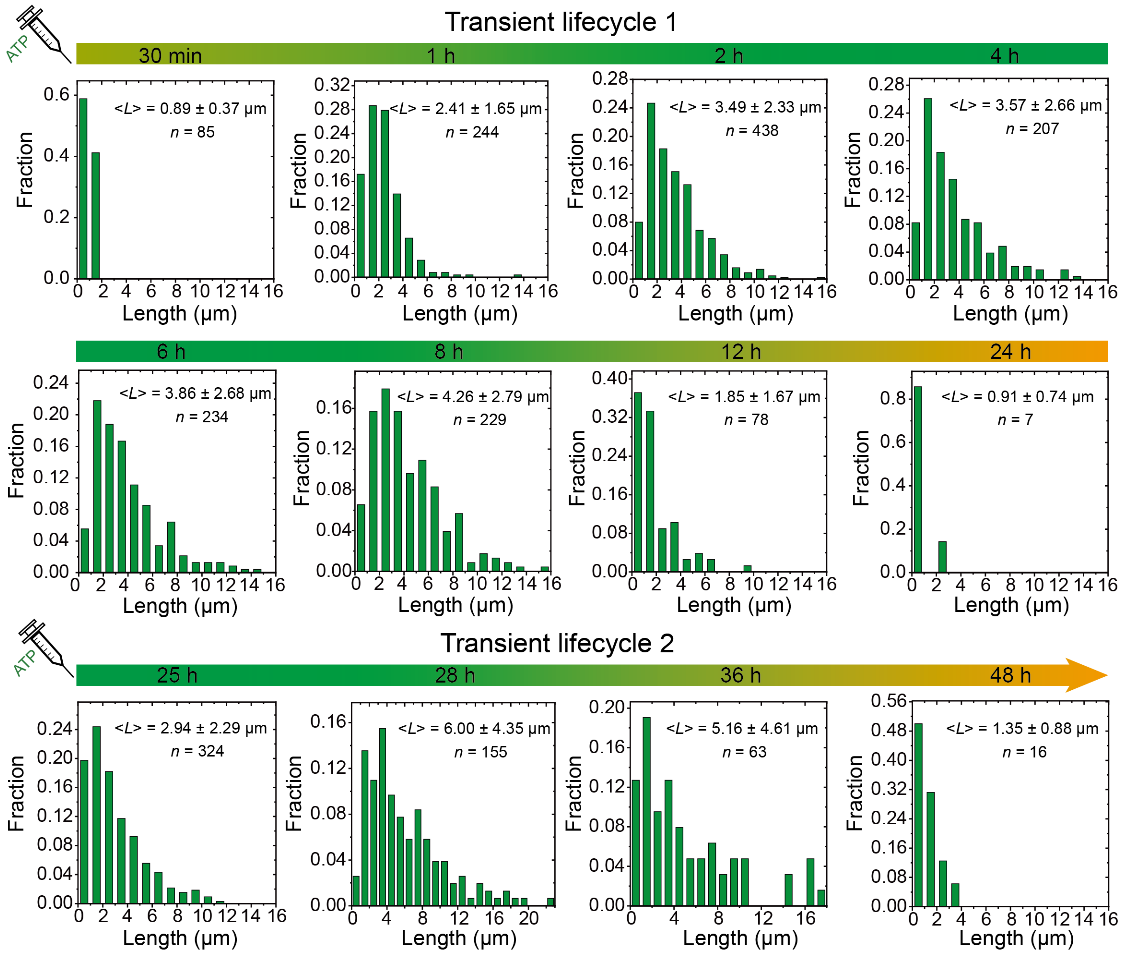

Supplementary Fig. 5. Histograms of nanotube length measured from CLSM images. Time-dependent length distribution of the ATP-fueled transient DNTs. <*L*> indicates mean nanotube length.

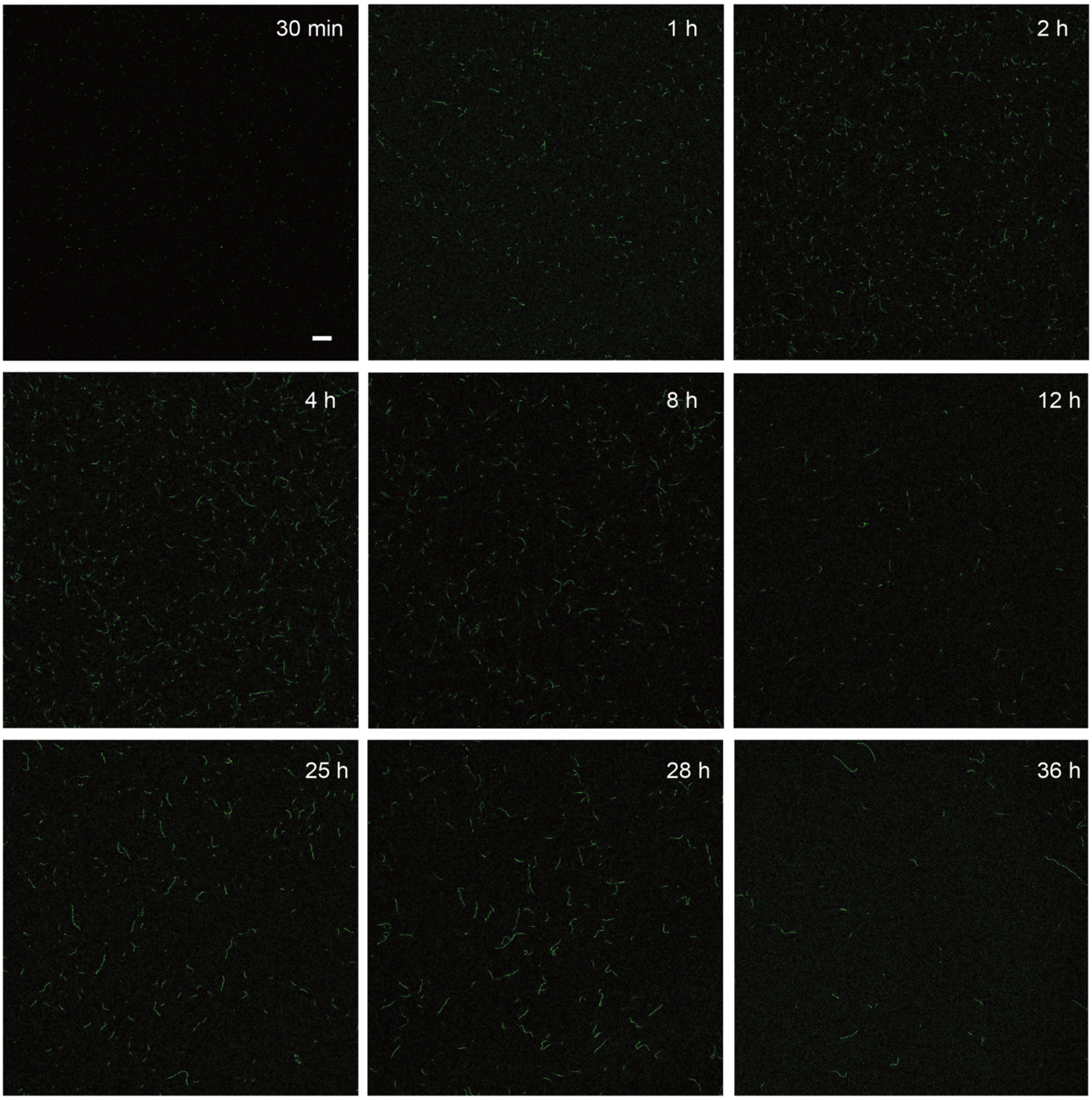

Supplementary Fig. 6. Zoomed-out CLSM images of ATP-fueled transient DNT self-assembly. Time-dependent representative CLSM images of ATP-fueled transient DNTs. Scale bar = 10 µm. Conditions: 25 °C, 0.5 µM Inactive tile 1 (0.5 µM tile + 1 µM Inhibitor), 20 µM Complex 1, 5 µM Substrate 1, 10 µM Input 1, 10 µM Input 2, 0.8 WU μL^{-1} T4 DNA ligase, 1.5 U μL^{-1} BsaI, and 40 µM ATP. At 24 h, another 40 µM ATP was added to initiate the second transient lifecycle.

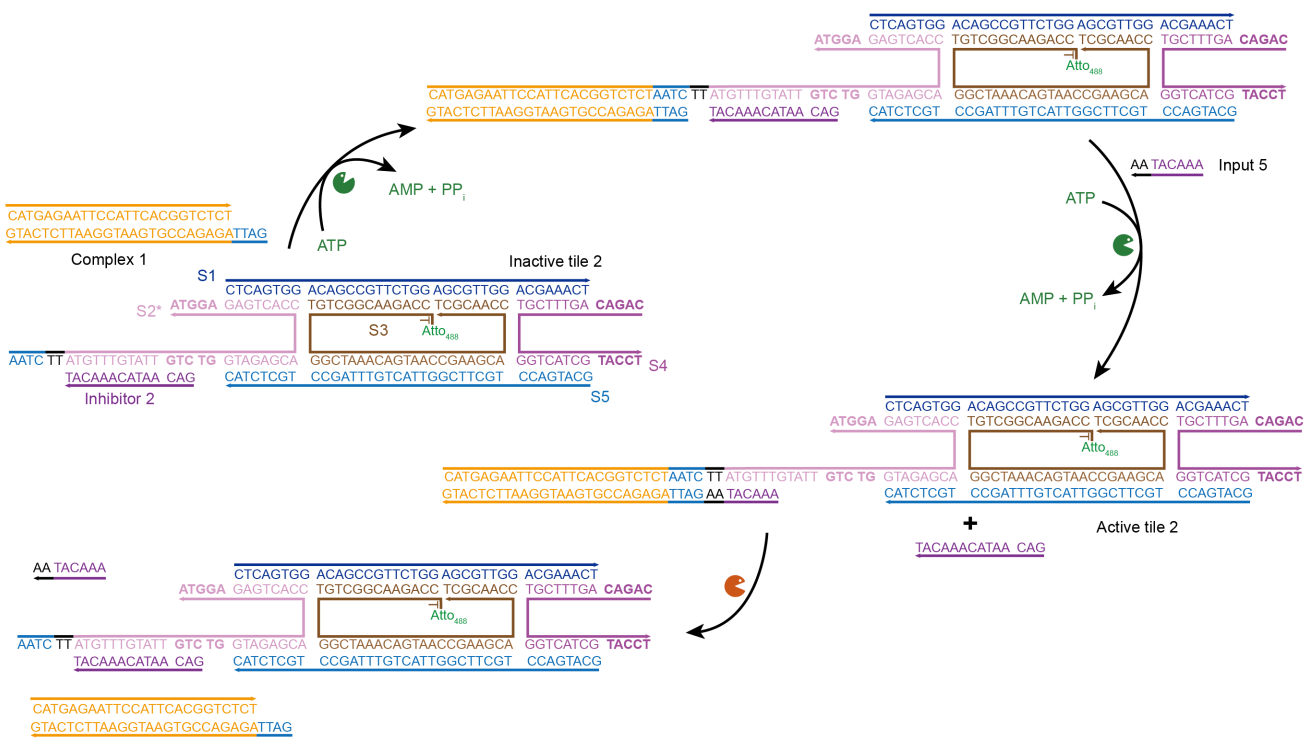

Supplementary Fig. 7. Illustration of temporal tile activation via direct ATP-powered transient ligation on tile. Schematic representation of Inactive tile 2 annealed from S 1, S 2*, S 3, S 4, S 5, and Inhibitor 2, and its activation by two consecutive steps ligation with Complex 1 and Input 5. The BsaI restriction can simultaneously deactivate the activated tile to regenerate Inactive tile 2, Complex 1, and Input 5.

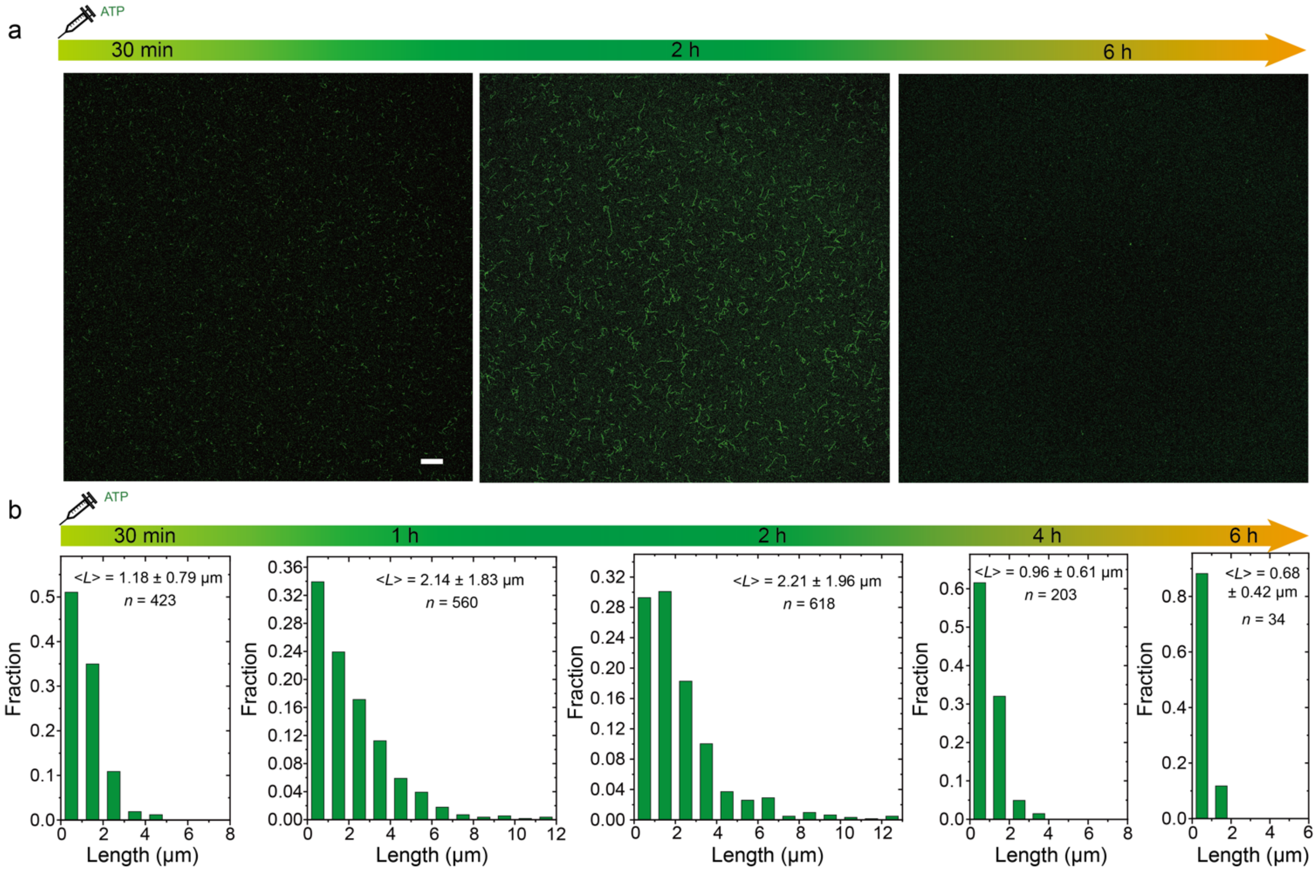

Supplementary Fig. 8. ATP-fueled transient DNT by direct ATP-powered transient ligation on assembling tile. (**a**) Time-dependent representative CLSM images of ATP-fueled transient DNTs. Scale bar = 10 μm. (**b**) Time-dependent length distribution of the ATP-fueled transient DNTs measured from CLSM images. <*L*> indicates mean nanotube length. Conditions: 25 °C, 1 μM Inactive tile 2 (1 μM tile + 1 μM Inhibitor), 5 μM Complex 1, 5 μM Input 5, 0.8 WU μL^{-1} T4 DNA ligase, 1.5 U μL^{-1} BsaI, and 8 μM ATP.

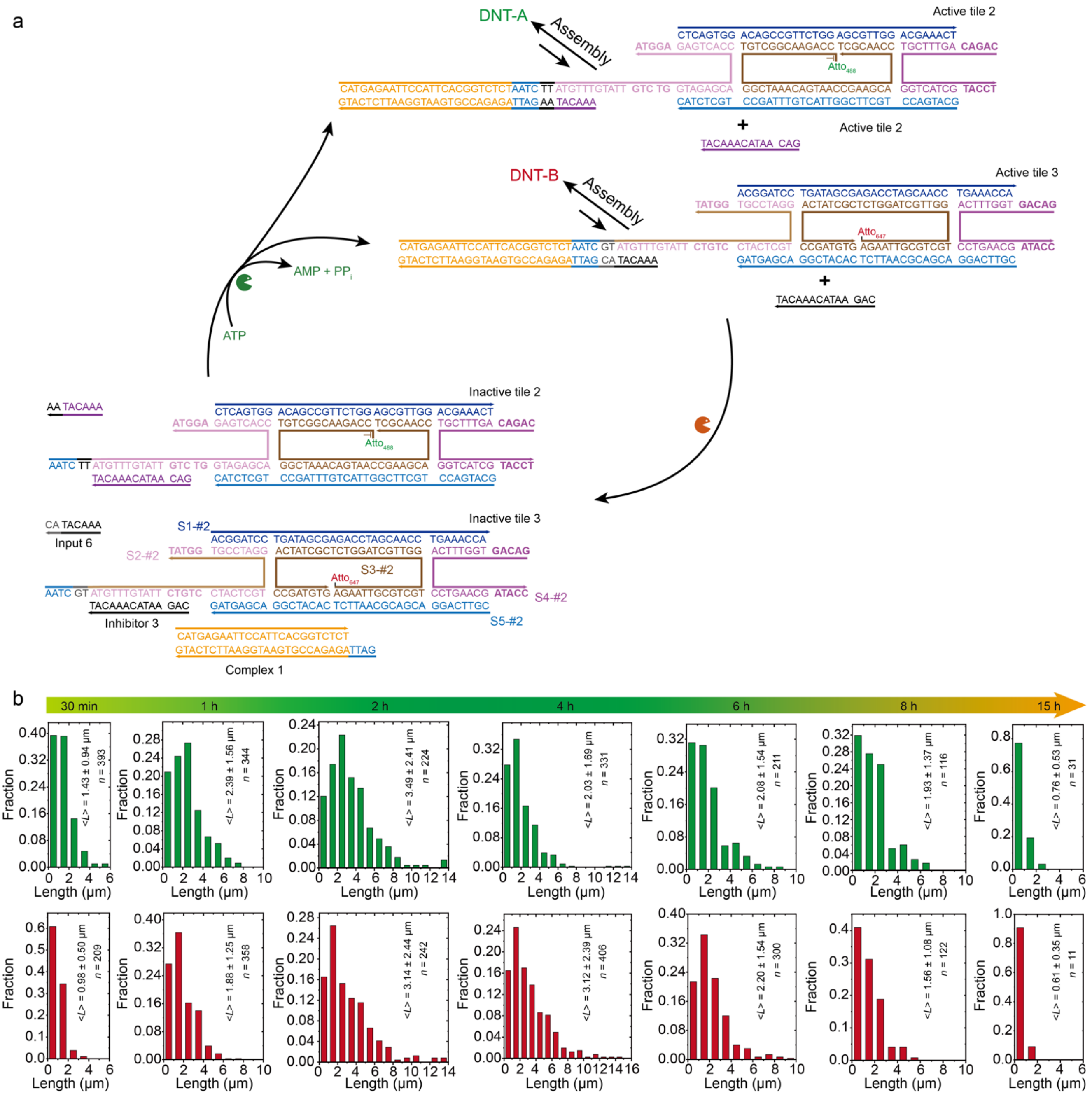

Supplementary Fig. 9. ATP-fueled transient self-sorting DNTs. (**a**) Illustration of ATP-fueled transient activation of Inactive tile 2 and Inactive tile 3 for the assemblies of DNT-A and DNT-B, respectively. Inactive tile 2 was annealed from S 1, S 2*, S 3, S 4, S 5, and Inhibitor 2. Inactive tile 3 was annealed from S 1-#2, S 2-#2, S 3-#2, S 4-#2, S 5-#2, and Inhibitor 3. (**b**) Histograms of nanotube length measured from CLSM images show the time-dependent length distribution of the ATP-fueled transient DNTs. The green and red histograms represent the nanotube length of DNT-A and DNT-B, respectively. <*L*> indicates mean nanotube length. Conditions: 25 °C, 1 µM Inactive tile 2 (1 µM tile + 1 µM Inhibitor), 1 µM Inactive tile 3 (1 µM tile + 1 µM Inhibitor) 10 µM Complex 1, 5 µM Input 5, 5 µM Input 6, 0.8 WU μL^{-1} T4 DNA ligase, 1.5 U μL^{-1} BsaI, and 16 µM ATP.
